# Modification of MTEA-Based Temperature Drift Error Compensation Model for MEMS-Gyros

**DOI:** 10.3390/s20102906

**Published:** 2020-05-21

**Authors:** Bing Qi, Fuzhong Wen, Fanming Liu, Jianhua Cheng

**Affiliations:** 1 College of Automation, Harbin Engineering University, Harbin 150001, China; hrblfm407@hrbeu.edu.cn (F.L.); ins_cheng@163.com (J.C.); 2 Southwest China Research Institute of Electronic Equipment, Chengdu 610036, China; wenfuzhong@163.com

**Keywords:** MEMS-gyros, temperature dependence, microstructure thermal effect analysis, TDE precise test based on heat conduction analysis, RBF ANN

## Abstract

The conventional temperature drift error (TDE) compensation model cannot decouple temperature dependence of Si-based materials because temperature correlated quantities (TCQ) have not been obtained comprehensively, and Micro-Electro-Mechanical System gyros’ (MEMS-gyros’) environmental adaptability is reduced in diverse, complicated conditions. The study presents modification of TDE compensation model of MEMS-gyros based on microstructure thermal effect analysis (MTEA). First, Si-based materials’ temperature dependence was studied in microstructure with thermal expansion effect and TCQ that determines the structural deformation were extracted to modify the conventional model, including temperature variation and its square. Second, a precise TDE test method was formed by analyzing heat conduction process between MEMS-gyros and thermal chamber, and temperature experiments were designed and conducted. Third, the modified model’s parameters were identified based on radical basis function artificial neural network (RBF ANN) and its performance was evaluated. Last, the conventional and modified models were compared in performance. The experimental results show MEMS-gyros’ bias stability was up to 10% of the conventional model, the temperature dependence of Si-based materials was decoupled better by the modified one and the environmental adaptability of MEMS-gyros was improved to expand their application in diverse complicated conditions.

## 1. Introduction

With the progress of science and technology, humans’ willingness to explore space and develop resources is increasing [1,2,3,4,5,6,7]. As is known, there are many rare resources in deep space, and there may even be an adaptive environment supporting life on the earth, and humans are attracted to go into action. Due to the extremely harsh environment in space, humans are unable to reach far. Instead, unmanned intelligent devices have been designed and used, such as all-weather Unmanned Aerial Vehicle (UAV) monitoring systems, lunar and Mars rovers, micro satellites, etc. [8,9,10,11,12]. Considering navigating or guiding unmanned intelligent devices to the target location safely, it is necessary to stabilize their attitudes. Gyros are indispensable to such navigation systems. Currently, unmanned intelligent devices have characteristics such as small size, low power consumption, incredible environmental adaptability—all of which require gyros to meet or even exceed stringent requirements. Base on this, resonator gyros have been invented and used. By measuring Coriolis force, carriers’ angular velocities are obtained, and their attitudes are measured. Table 1 shows the comparison between the resonator gyros.

Considering the accuracy, cost, size, stability and reliability comprehensively, Micro-Electro-Mechanical System gyros (MEMS-gyros) are easy to apply as well as precise to navigate and guide, and this is the best choice to unmanned intelligent devices. They are used to measure the attitude of unmanned intelligent devices’, which plays an important role in their stability and safety. For example, when unmanned rovers cruise on a planet with a complex terrain, measuring their stability in real time can predict if overturning and sinking is imminent, and alert them to take solutions to avoid the coming risk, such engaging an emergency brake or changing course. In addition, it can warn unmanned aerial vehicles of unsafe flight situations to avoid crash. However, MEMS-gyros are fabricated with temperature dependent Si-based materials, and their physical properties change as ambient temperature varies. Ambient temperature in space is about −180 °C–130 °C, and inevitably TDE come into play to reduce MEMS-gyros’ stability. Taking the accuracy of MEMS-gyro into account (±0.00875°/s) for example, when ambient temperature varies by 10 °C, its TDE is about 0.7°/s [13]. This results in attitude errors, velocity errors and heading errors accumulating over time, and some wrong references are given to unmanned intelligent devices. Maybe, they take some improper solutions to aggravate the dangerous situations. Hence, TDE restrict MEMS-gyros use in diverse complicated conditions; eliminating TDE plays a very important role in unmanned intelligent devices’ stability and safety. Decoupling temperature dependence of Si-based materials becomes the key to eliminate TDE and improve the environmental adaptability of MEMS-gyros [14,15].

To solve these problems, Masako Tanaka et al. analyzed the key factors affecting MEMS-gyros’ stability in depth and determined that structural consistency influenced driving frequency of driving circuit and combs stability of sensing circuit [16]. Liu et al. studied the effect of temperature variation on MEMS-gyros and especially the process that structural consistency changed the driving frequency [17]. They showed MEMS-gyros’ structural consistency lied in temperature dependence of Si-based materials in essence whose permanence is induced by constant ambient temperature. Hence, temperature control is optional to maintain structural consistency [18]. However, it needs high-power equipment to stabilize ambient temperature at the target, which conflicts with MEMS-gyros’ merits mentioned. Instead, digital compensation correction is an alternative, like Least Square Method (LSM), Kalman filter, artificial neural network. Jiaying Du et al. compared LSM, Kalman filter and digital filter in TDE compensation and studied their merits of their accuracy in real time [19]. TDE are estimated by them with ambient temperature. Kalman filter has highest accuracy, but worst real time due to its high dimensional matrixes and complex functions. To improve its real time further, Rita Fontanella et al. built a compensation model with augmented state Kalman filter. Its accuracy is maintained and its real time is improved by optimizing internal structure [20]. Markedly, Kalman filter depends on hardware resources to support its high accuracy and inevitably increases costs. Instead, LSM has the merits of estimating TDE accurately and quickly [21,22,23,24]. Igor P. Prikhodko et al. established LSM linear model with quality factor and ambient temperature [25]. Using quality factor, ambient temperature is estimated approximately and scale factor—as well as bias—is estimated to compensate TDE in real time. Scale factor and bias are up to 700 ppm and 2°/h. However, LSM is weakly able to restrain random error, so it needs a restraining method. Fuchao Liu and Hao Zheng et al. proposed an integrated model based on LSM and Kalman filter [26,27]. Kalman filter restrains random error in sample data and LSM estimates TDE with ambient temperature. Considering that Kalman filter only restrains random error, but does not calculate TDE, its real time is harmed, but in an allowable range. Improving the accuracy of LSM relies on increasing the fitting order and the higher the fitting order is the greater accuracy appears. However, it is unilateral that greater accuracy only relies on increasing the fitting order, which leads to over-compensation even real time reduction. Based on a number of test data, Bourgeteau et al. concluded complex nonlinearity appeared between ambient temperature and TDE, and describing it accurately was a prerequisite to TDE compensation accuracy. It also showed LSM could not increase TDE compensation accuracy further [28]. BP-ANN was introduced to describe the nonlinearity accurately [29,30,31]. It takes ambient temperature as the input and TDE as the output. By training BP-ANN, TDE compensation model is established after the compensation requirements are met. However, there may be local minimums which worsens its generalization ability and compensation accuracy in global scope. More important, TCQ exciting TDE should be figured out clearly and completely, which offers precise references to describe TDE. Jan K. Bekkeng et al. researched TCQ in depth and revealed that ambient temperature variation was a key factor to the structural deformation of MEMS-gyros [32]. Using ambient temperature variation, MEMS-gyros’ bias stability was improved and increased by more than one order of magnitude.

Improving the performance of TDE compensation is determined by three factors: TCQ exciting TDE, accurately describing the complex nonlinearity between TCQ and TDE, testing TDE precisely to identify the compensation model’s parameters. Hence, structural deformation of Si-based materials in MEMS-gyros is analyzed precisely with thermal expansion theory to extract TCQ comprehensively. With heat conduction analysis, TDE precise test method forms and its key parameters are deduced, including temperature jump interval and its period, and temperature experiments are conducted. A modified RBF ANN-based TDE compensation model is established and its parameters are identified precisely. It can estimate TDE more accurately and decouple Si-based materials’ temperature dependence effectively, and that increases MEMS-gyros’ stability and improves the environmental adaptability. It is significant to expand MEMS-gyros’ application in diverse complicated conditions and guarantees the safety and stability of unmanned intelligent devices and similar smart systems.

This article is organized as follows: in Section 2, TCQ of MEMS-gyros are extracted to form a modified TDE compensation model. Section 3 demonstrates the entire scheme for the modified model based on input-modified RBF ANN and shows the collected data utilized to train the input-modified RBF ANN and the experimental results of the MEMS-gyros-compensated temperature experiments. Section 4 evaluates TDE compensation performances of the proposed scheme compared to the previous conventional schemes. Section 5 presents the conclusions and benefits of the novel method.

## 2. Modification of TDE Compensation Model for MEMS-Gyros

### 2.1. Conventional TDE Compensation Model

**Figure 1:**
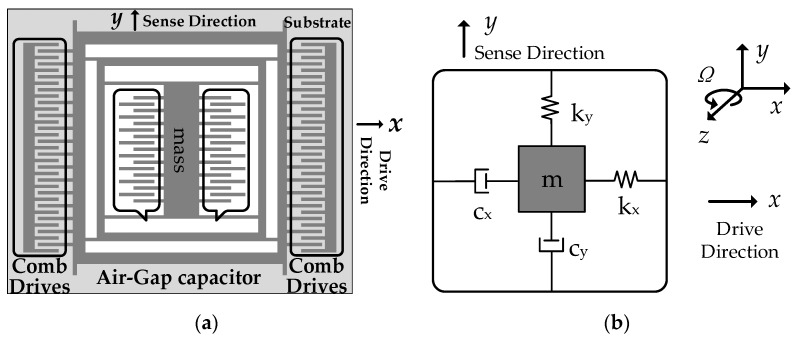
Functional schematic diagram of MEMS-gyros. (**a**) hardware design; (**b**) system schematic.

MEMS-gyros are a kind of miniaturized devices manufactured with Si-based materials. They are mainly composed of the mass, the driving circuit, the sensing circuit and the substrate. With a series of procedure, including design, process, manufacture, measure and control, all of components are combined and integrated as a micromachining unit. Figure 1 shows the hardware design diagram of MEMS-gyros and their system schematic diagram [17].

**Figure 2:**
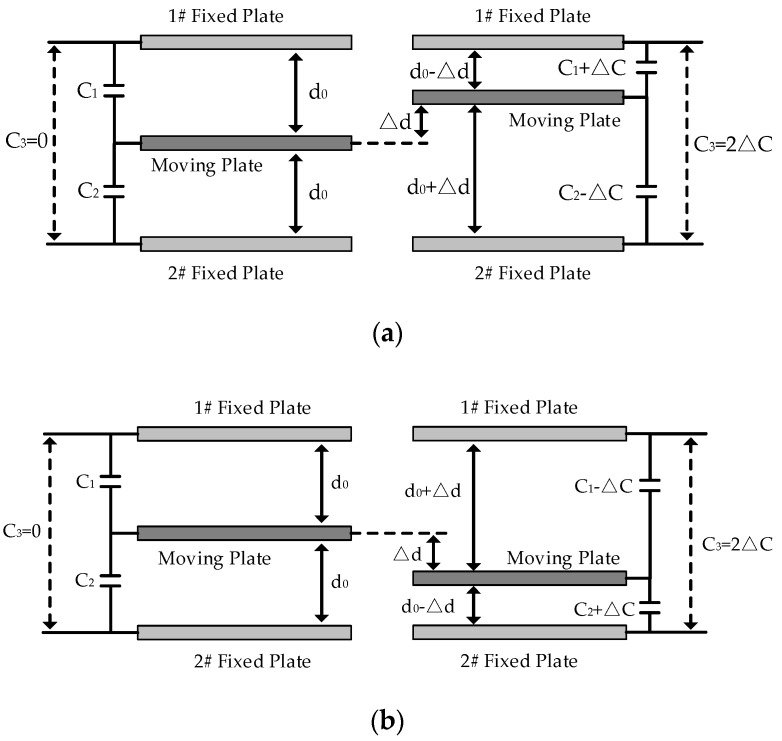
State diagram of the combs before and after rotation. (**a**) clockwise; (**b**) counter-clockwise.

Where $k_{x}$ and $k_{y}$ are stiffness coefficients of springs in driving direction *x*-axis and sensing direction *y*-axis, $c_{x}$ and $c_{y}$ are damping coefficients in *x*-axis and *y*-axis. With the help of the driving circuit, the mass m vibrates along *x*-axis under sinusoidal voltage with frequency $\omega_{d}$. When MEMS-gyros rotate along *z*-axis at angular velocity Ω, Coriolis force causes an displacement along *y*-axis. Technically, combs of sensing circuit can be abstracted as plate capacitors composed of moving plates and fixed plates. Any carrier’s angular velocity is obtained by measuring the capacitance variation in *y*-axis. Figure 2 shows the state diagram of combs before and after clockwise or counter clockwise rotation.

Based on plate capacitors’ definition, $C_{3}$ between 1# fixed plate and 2# fixed plate can be obtained:

(1) $$C_{3}=\left|C_{1}-C_{2}\right|=\left|\frac{\varepsilon }{4\pi k}\frac{S_{0}}{\left(d_{0}+\Delta d\right)}-\frac{\varepsilon }{4\pi k}\frac{S_{0}}{\left(d_{0}-\Delta d\right)}\right|=2\Delta C$$

Hence, angular velocity of the carrier can be obtained by measuring the capacitance variation. However, due to temperature dependence of Si-based materials, the stiffness of the driving circuit and the sensing circuit changes with ambient temperature. In particular, the stiffness is critical to the resonant frequencies in driving direction *x*-axis. According to elastic modulus formula, the elastic modulus variation of Si-based materials with ambient temperature can be described as follows:

(2) $$E\left(T\right)=E\left(T_{0}\right)\left[1-k\left(T-T_{0}\right)\right]$$

where E(T) and $E\left(T_{0}\right)$ are the elastic modulus of Si-based materials at temperature T and $T_{0}$ separately, k is the elastic coefficient of Si-based materials and k=70 ppm, $T_{0}=300\;K$. Based on that, the spring stiffness coefficient is expressed as follows:

(3) $$K\left(T\right)=K\left(T_{0}\right)\left[1-k\left(T-T_{0}\right)\right]$$

where K(T) and $K\left(T_{0}\right)$ are the stiffness coefficients at temperature T and $T_{0}$. Hence, resonant frequency in *x*-axis $\omega_{x}\left(T\right)$ can be induced as follows:

(4) $$\omega_{x}\left(T\right)=\sqrt{K\left(T_{0}\right)\left[1-k\left(T-T_{0}\right)\right]/m_{x}}$$

where $m_{x}$ is the effective mass in driving mode. The resonant amplitude $u_{\max}\left(T\right)$ and its phase $\alpha_{x}\left(T\right)$ can be deduced as follows [17]:

(5) $$\begin{array}{l} u_{\max}\left(T\right)=\frac{F_{e}m_{x}}{\sqrt{{\left\{{\left[\omega_{x}\left(T_{0}\right)\right]}^{2}{\left[1-k\left(T-T_{0}\right)/2\right]}^{2}-\omega_{d}^{2}\right\}}^{2}+{\left\{\omega_{x}\left(T_{0}\right)\left[1-k\left(T-T_{0}\right)/2\right]\omega_{d}/Q_{x}\right\}}^{2}}}\\ \alpha_{x}\left(T\right)=\arctan\frac{\omega_{x}\left(T_{0}\right)\left[1-k\left(T-T_{0}\right)/2\right]\omega_{d}}{Q_{x}\omega_{x}{\left(T_{0}\right)}^{2}{\left[1-k\left(T-T_{0}\right)/2\right]}^{2}-\omega_{d}^{2}} \end{array}$$

where $F_{e}$ is the amplitude of the external driving force in *x*-axis, $Q_{x}=k_{x}/c_{x}\omega_{x}$. From Equation (5), the resonant amplitude and its phase are determined by ambient temperature T and its reference $T_{0}$, which results in the resonant frequency instability as ambient temperature variation ΔT and $\Delta T=T_{1}-T_{0}$. Unavoidably, it introduces TDE in the output of MEMS-gyros. Hence, the conventional TDE compensation model considers ambient temperature variation as TCQ, and it is shown as follows:

(6) $$\Delta E_{MEMS}=f(\Delta T)$$

### 2.2. Modified TDE Compensation Model

As it is known, although the stability of MEMS-gyros is directly determined by the resonant frequency in the driving circuit, it is not the only key factor to TDE. Furthermore, it cannot be ignored that the stiffness of the sensing circuit changes with ambient temperature as well, and the measuring error of the capacitance in sensing circuit should be reconsidered as another key factor. Taking the combs in sensing circuit for example, due to temperature dependence of Si-based materials, structural deformation of the combs appears in three-dimensional space as ambient temperature varies and the structural consistency changes. Assuming that ambient temperature is T and the angular velocity of carriers is ω, MEMS-gyros under diverse conditions were simulated as follows.

• $\begin{array}{l} T=T_{0} \end{array}$ and ω=0

**Figure 3:**
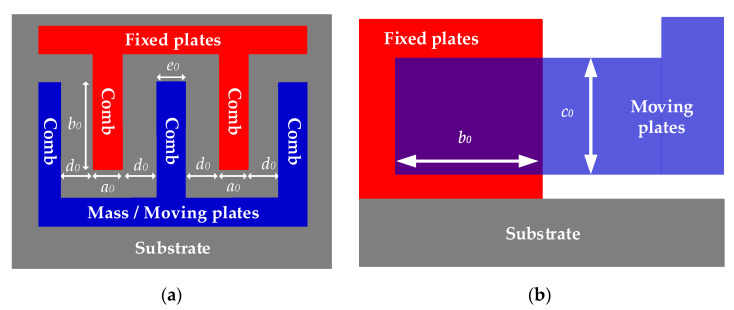
Combs of moving plates and fixed plates when $T=T_{0}$ and ω=0. (**a**) top view; (**b**) side view.

When ambient temperature is $T_{0}$ constantly, the internal structure of MEMS-gyros is stable. When the carriers does not rotate and ω=0, moving plates and fixed plates are in a balanced state in ideal case. Figure 3 shows the combs of moving plates and fixed plates when ω=0.

Where $a_{0}$ is the thickness of the combs of fixed plates,$b_{0}$ is the length of the overlap between moving plates and fixed plates, $c_{0}$ is the width of the overlap between moving plates and fixed plates, $d_{0}$ is the distance between the combs of moving plates and fixed plates, $e_{0}$ is the thickness of the combs of moving plates. As shown in Figure 3, assuming that the combs are fabricated in an ideal case, then the capacitance measured in sensing circuit is as follows:

(7) $$C_{3}=\left|C_{1}-C_{2}\right|=\left|\frac{\varepsilon }{4\pi k}\frac{b_{0}c_{0}}{d_{0}}-\frac{\varepsilon }{4\pi k}\frac{b_{0}c_{0}}{d_{0}}\right|=0$$

• $T=T_{0}$ and $\omega =\omega_{0}\left(\omega_{0}\neq 0\right)$

**Figure 4:**
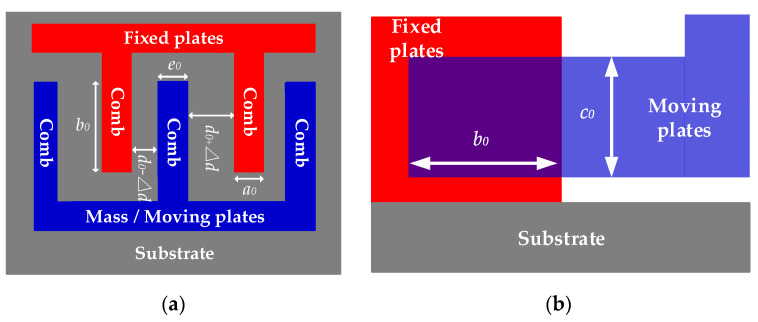
Combs of moving plates and fixed plates when $T=T_{0}$ and $\omega =\omega_{0}$. (**a**) top view; (**b**) side view.

When ambient temperature stays still $T_{0}$ and the carrier rotates at the angular velocity of $\omega =\omega_{0}$, Coriolis force acts on the mass m and a displacement appears in *y*-axis. Moving plates as well as fixed plates are in an unbalanced state, which changes the capacitance in the sensing circuit. Figure 4 shows the change of the combs of moving plates and fixed plates when $\omega =\omega_{0}$.

From Figure 4, the combs of moving plates displace from the balanced state and according to Equation (1) the capacitance measured in the sensing circuit is expressed as follows:

(8) $$C_{3}=\left|C_{1}-C_{2}\right|=\left|\frac{\varepsilon }{4\pi k}\frac{b_{0}c_{0}}{d_{0}-\Delta d}-\frac{\varepsilon }{4\pi k}\frac{b_{0}c_{0}}{d_{0}+\Delta d}\right|=2\times \frac{\varepsilon b_{0}c_{0}}{4\pi k}\frac{\Delta d}{\left(d_{0}-\Delta d\right)\left(d_{0}+\Delta d\right)}$$

• $T=T_{1}\left(T_{1}\neq T_{0}\right)$ and $\omega =\omega_{0}\left(\omega_{0}\neq 0\right)$

**Figure 5:**
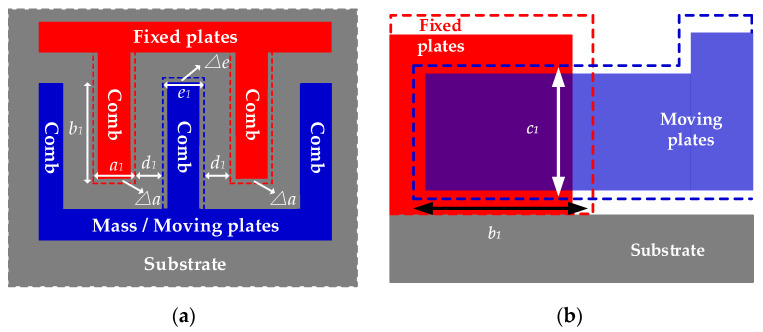
Deformation of the combs when $T=T_{1}$ and ω=0. (**a**) top view; (**b**) side view.

When $T=T_{1}$, the combs deform in three dimensions as ambient temperature varies because of temperature dependence of Si-based materials, including expanding or contracting. Figure 5 shows the deformations before and after temperature variation when ω=0.

Where $a_{1}$ is the thickness of the combs of fixed plates after deformation, $b_{1}$ is the length of the overlap between moving plates and fixed plates after deformation, $c_{1}$ is the width of the overlap between moving plates and fixed plates after deformation, $d_{1}$ is the distance between the combs of moving plates and fixed plates after deformation, $e_{1}$ is the thickness of the combs of moving plates after deformation, Δa is the thickness variation of the combs of fixed plates after deformation, Δe is the thickness variation of the combs of moving plates after deformation. According to thermal expansion theory, three-dimensional sizes of the combs shown in Figure 5 after deforming are shown as follows:

(9) $$\left\{ \begin{array}{c} a_{1}=a_{0}\left[\alpha_{T}(T_{1}-T_{0})+1\right]=a_{0}(\alpha_{T}\Delta T+1)\\ c_{1}=c_{0}\left[\alpha_{T}(T_{1}-T_{0})+1\right]=c_{0}(\alpha_{T}\Delta T+1)\\ e_{1}=e_{0}\left[\alpha_{T}(T_{1}-T_{0})+1\right]=e_{0}(\alpha_{T}\Delta T+1) \end{array}\right.$$

where $\alpha_{T}$ is the thermal expansion coefficient of Si-based materials, ΔT is ambient temperature variation and $\Delta T=T_{1}-T_{0}$. Under the excitation of TCQ, the combs of moving plates and fixed plates deform, respectively at the same time. In Figure 5b, assuming that the combs of moving plates deform in transverse left direction and its transverse length expands or contracts by Δe, the combs of fixed plates deform in transverse right direction and its transverse length also expands or contracts by Δa because fixed plates and moving plates have the same degrees of freedom for deformation. Hence, the total transverse length of the overlap of combs $b_{1}$ can be described as follows:

(10) $$b_{1}=2b_{0}\left[\alpha_{T}(T_{1}-T_{0})+1\right]=2b_{0}(\alpha_{T}\Delta T+1)$$

In addition, from Figure 5a, the transverse extension of the combs of fixed plates Δa and the transverse extension of the combs of moving plates Δe are, respectively shown as follows:

(11) $$\begin{array}{l} \Delta a=a_{1}-a_{0}=a_{0}\alpha_{T}\Delta T\\ \Delta e=e_{1}-e_{0}=e_{0}\alpha_{T}\Delta T \end{array}$$

Because the combs deform in transverse left direction and in transverse right direction, the distance between the combs of moving plates and fixed plates after deformation $d_{1}$ is expressed:

(12) $$d_{1}=d_{0}-\frac{\Delta a}{2}-\frac{\Delta e}{2}=d_{0}-\frac{\alpha_{T}\Delta T\left(a_{0}+e_{0}\right)}{2}$$

**Figure 6:**
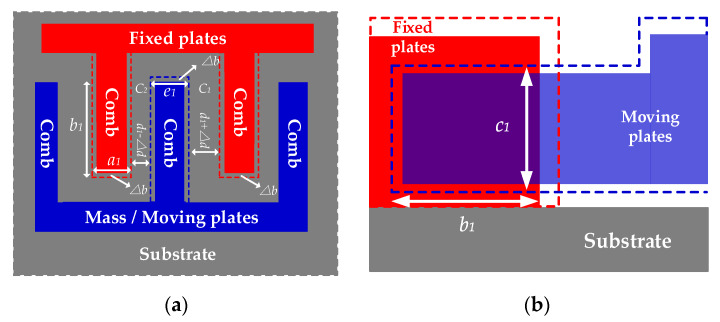
Deformation of the combs when $T=T_{1}$ and $\omega =\omega_{0}$. (**a**) top view; (**b**) side view.

Given that the fabricated MEMS-gyros have firm structure, the mass has the same displacement under the same Coriolis force when the carriers rotate at the same angular velocity $\omega =\omega_{0}$. That means the relative distance between the mass’ center when the carriers rotate at ω=0 and $\omega =\omega_{0}$ still stay constant. Figure 6 shows the deformation of the combs when $\omega =\omega_{0}$ and $T=T_{1}$.

According to Equation (1), the capacitance measured in the sensing circuit is shown as follows:

(13) $$C_{3}{}^{\prime }=\left|C_{1}{}^{\prime }-C_{2}{}^{\prime }\right|=\left|\frac{\varepsilon }{4\pi k}\frac{b_{1}c_{1}}{d_{1}-\Delta d}-\frac{\varepsilon }{4\pi k}\frac{b_{1}c_{1}}{d_{0}+\Delta d}\right|=\left|2\times \frac{\varepsilon b_{1}c_{1}}{4\pi k}\frac{\Delta d}{\left(d_{1}-\Delta d\right)\left(d_{1}+\Delta d\right)}\right|$$

Then, from Equations (9), (10) and (12), we obtain:

(14) $$C_{3}{}^{\prime }=\left|\frac{2\varepsilon \Delta d2b_{0}c_{0}}{4\pi k}\times \frac{{\left(\alpha_{T}\Delta T+1\right)}^{2}}{d_{1}{}^{2}-\Delta d^{2}}\right|=\left|\frac{2\varepsilon \Delta d2b_{0}c_{0}}{4\pi k}\times \frac{4{\left(\alpha_{T}\Delta T+1\right)}^{2}}{4d_{0}{}^{2}+\alpha_{T}{}^{2}\Delta T^{2}{\left(a_{0}+e_{0}\right)}^{2}-4d_{0}\alpha_{T}\Delta T\left(a_{0}+e_{0}\right)-{\left(2\Delta d\right)}^{2}}\right|$$

According to Equations (7) and (14), then the capacitance error $\Delta C_{E}$ can be described:

(15) $$\begin{array}{cc} \Delta C_{E} & =C_{3}{}^{\prime }-C_{3}=\frac{2\varepsilon b_{0}c_{0}\Delta d}{4\pi k}\times \frac{2{\left(\alpha_{T}\Delta T+1\right)}^{2}}{d_{1}{}^{2}-\Delta d^{2}}-\frac{2\varepsilon b_{0}c_{0}}{4\pi k}\frac{\Delta d}{\left(d_{0}-\Delta d\right)\left(d_{0}+\Delta d\right)}\\ & =\frac{2\varepsilon \Delta db_{0}c_{0}}{4\pi k\left(d_{0}{}^{2}-\Delta d^{2}\right)}\times \frac{\alpha_{T}{}^{2}\left[8d_{0}{}^{2}-{\left(a_{0}+e_{0}\right)}^{2}-8\Delta d^{2}\right]\Delta T^{2}+4\alpha_{T}\left[4d_{0}{}^{2}+\left(a_{0}+e_{0}\right)d_{0}-4\Delta d^{2}\right]\Delta T+4d_{0}{}^{2}-4\Delta d^{2}}{\left[\alpha_{T}{}^{2}{\left(a_{0}+e_{0}\right)}^{2}\Delta T^{2}-4\alpha_{T}\left(a_{0}+e_{0}\right)d_{0}\Delta T+4d_{0}{}^{2}-4\Delta d^{2}\right]} \end{array}$$

From Equation (15), the capacitance error in sensing circuit is relevant to ambient temperature variation ΔT and its square $\Delta T^{2}$. Moreover, from Equation (6), ambient temperature variation is the key factor to the resonant frequency and important to TDE. Hence, ΔT and $\Delta T^{2}$ are the critical references to compensate TDE accurately. Hence, the modified TDE compensation model is established:

(16) $$\Delta E_{MEMS}=f(\Delta T,\Delta T^{2})$$

## 3. Design of Modified TDE Compensation Model

Implementation of the modified TDE compensation model for MEMS-gyros is a priori process, and a mathematical model with perfect structure and clear parameters is gradually established by analyzing the sufficient experimental data which are tested and obtained beforehand. Hence, precise test for TDE is necessary and essential to the modified TDE compensation model, and the parameters identification are an important guarantee to its implementation.

### 3.1. Design of Precise Test for Temperature Drift Error

According to Equation (16), the modified TDE compensation model uses ΔT and $\Delta T^{2}$ as the model inputs and TDE as the model output. Its accuracy depends on how precisely TDE can be accurately described with TCQ and that illustrates testing TDE and TCQ precisely is a prerequisite to the accuracy of the modified TDE compensation model. As it is known, TDE $\Delta E_{MEMS}$ consists of bias error, trend error and random error and they are expressed as follows:

(17) $$\Delta E_{MEMS}=E_{bias}+E_{trend}+E_{random}$$

where $E_{bias}$ is bias error which is a type of fixed deviation between the measured value of the angular velocity of the carriers and its theoretical value, $E_{trend}$ is trend error which is a type of linear or nonlinear deviation as ambient temperature varies, $E_{random}$ is random error which is a series of small random fluctuations of some related factors and mutually compensated in the long term. $E_{bias}$ and $E_{trend}$ account for most part of TDE, and it is possible to estimate and compensate accurately them due to their definite law and expression. When MEMS-gyros are manufactured, their environmental adaptability are unalterable. From the datasheets, their TDE can be grossly described as follows:

(18) ΔE=αΔT+βΔT

where ΔE are roughly estimated values of TDE, α is MEMS-gyros’ character named “Zero-rate level change vs. temperature”, β is MEMS-gyros’ character named “Sensitivity change vs. temperature”, $\Delta T=T-T_{0}$, and $T_{0}$ is the referenced ambient temperature. Theoretically, ΔE stands for part of TDE and is smaller than the true value of TDE in amplitude, so we can obtain as follows:

(19) $$\Delta E\leq \Delta E_{MEMS}$$

Because MEMS-gyros’ sensitivity determines the measured minimum of the angular velocity of the carriers $\Delta E_{S}$, it is very possible that TDE appears greater than MEMS-gyros’ sensitivity when ambient temperature jumps rapidly, even completely submerging the actual angular velocity of the carriers. In that case, the amplitude of $E_{random}$ also increases as TDE and greater than the actual angular velocity reference, then the deviation of the angular velocity reference is introduced. Based on that, it will make the angular velocity of the carrier be measured inaccurately and reduce the credibility of the angular velocity reference from the rate table. In order to test TDE accurately, TDE should be less than $\Delta E_{S}$, which is $\Delta E_{MEMS}\leq \Delta E_{S}$. Hence, according to Equation (18), we obtain:

(20) $$\Delta T\leq \frac{\Delta E_{S}}{\left|\alpha \right|+\left|\beta \right|}$$

**Figure 7:**
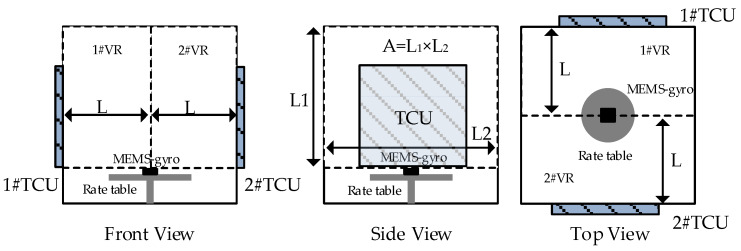
Schematic diagram of MEMS-gyros installed on the rate table inside the thermal chamber.

Hence, a precise test for TDE should meet the equation given in Equation (20), which defines ambient temperature variation in precise test for TDE. In that case, MEMS-gyros are installed on a precise rate table in thermal chamber. At present, the thermal chambers adopt the structural design of front-door opening and temperature control unit (TCU) arranged on left and right sides, and a precise rate table locates in the center of the thermal chamber. Moreover, it adopts closed insulation design to prevent heat leakage to form high or low temperature condition. Figure 7 shows the schematic diagram of MEMS-gyros installed on the rate table inside the thermal chamber.

Considering the universality and the reliability of the test results, the following key operations should be paid more attention:

• Heat conduction measures

In order to control temperature gradient effect perfectly to improve the real time in the test, heat conduction measures should be taken to ensure ambient temperature in the thermal chamber is completely the same as that of MEMS-gyros, which reduces heat conduction delay effect.

• Precise temperature measurement system

To obtain TCQ more accurately, precise temperature measurement system is utilized, and temperature sensors are installed closely at the surface of MEMS-gyros. The measurement accuracy of precise temperature measurement system should be more than 2 times more precise than ambient temperature variation and its measurement frequency should be higher than the output frequency of MEMS-gyros to reserve the margin for the accuracy of the test results.

• Reasonable temperature control sequence

According to heat conduction theory, it takes some time to transfer heat from place A to place B. Assuming that temperature at place A varies in a sequence $T_{A}=\left[T^{\prime },T^{\prime \prime },T^{\prime \prime \prime }\right]$, after transferring in time sequence $t=\left[t_{1},t_{2}\right]$, temperature at place B varies in a sequence $T_{B}=\left[T^{\prime },T^{\prime \prime },T^{\prime \prime \prime }\right]$. That points out it takes $t_{1}$ and $t_{2}$ to transfer the heat from place A to place B completely. During the period $t_{1}$, temperature at place B is between $T^{\prime }$ and $T^{\prime \prime }$. At that time, if temperature at place A varies to $T^{\prime \prime \prime }$, temperature at place B will finally vary to $T^{\prime \prime \prime }$. However, temperature at place B never stays stable at $T^{\prime \prime }$ for a while. If that appears in precise test for TDE, TDE of MEMS-gyros is not tested accurately. Hence, a reasonable temperature control sequence that keeps transferring heat completely and stalely is an important guarantee for testing TDE accurately.

As shown in Figure 7, the internal space of the thermal chamber is artificially divided into two independent spaces with identical physical characteristics, 1#VR and 2#VR. TCUs control ambient temperature in the thermal chamber through its inner wall. Given that two independent spaces are cubes with size of $L\times L_{1}\times L_{2}$ mm, heat from TCUs uniformly transfers to the joint of the independent spaces along length L which is perpendicular to the inner wall. The farther the location is away from the inner wall, the longer heat from TCUs transfer. The joint of independent spaces is the last area where ambient temperature stays stable. From Thermal Conductivity Formula, we can obtain:

(21) $$k=\frac{Q}{t_{s}}\frac{L}{A\Delta T}$$

where Q is the conducted heat, $t_{s}$ is the time for heat conduction, L is the length of heat conduction, A is the section area of heat conduction, ΔT is ambient temperature variation. From the calculation formula of specific heat capacity, the heat heating the independent spaces uniformly can be expressed:

(22) Q=CmΔT

where C is specific heat capacity of air inside thermal chamber,m is the total mass of air in a closed state. Substituting Equation (22) into Equation (21), a new equation can be obtained as follows:

(23) $$k=\frac{Cm\Delta T}{t_{s}}\frac{L}{A\Delta T}=\frac{Cm}{t_{s}}\frac{L}{A}$$

The time for heat conduction uniformly in the independent spaces can be deduced:

(24) $$t_{s}=\frac{Cm}{k}\frac{L}{A}=\frac{C\rho v}{k}\frac{L}{A}=\frac{C\rho LA}{k}\frac{L}{A}=\frac{C\rho L^{2}}{k}$$

where ρ is air density in the thermal chamber. From Equation (24), the time for heat conduction from the inner wall of the thermal chamber to the center of the rate table can be calculated precisely. To guarantee that both of independent spaces are heated uniformly, the period $t_{p}$ when the current temperature control target goes to the next one can be expressed:

(25) $$t_{s}\leq t_{p}$$

**Figure 8:**
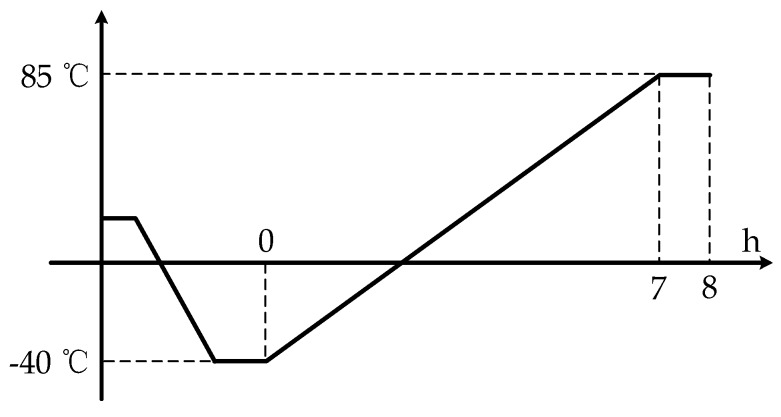
Flow chart of temperature experiment.

Hence, Equation (20) is taken as a reference to temperature jump interval and Equation (25) is taken as a reference to temperature jump period. Based on the discussion, L3GD20 manufactured by ST company is tested for TDE. From the datasheet, $\Delta E_{S}=8.75\;\mathrm{mdps}/\mathrm{digit}$, α=±0.03 dps/°C, its temperature range is −40 °C~85 °C. After dimensional transformation, β=2%×FS/[(85°C)−(−40 °C)]=0.04 dps/°C. Using Equation (20), temperature jump interval ΔT is found as 0.125 °C. Given that accurately testing TDE and simplifying test steps, it is essential to set ΔT=0.1 °C. Using thermal chamber SET-Z-021 to test L3GD20, C=1.005 kJ/(kg×K), k=0.0267 W/m°C, L=0.6 m, $\rho =1.293\;{\mathrm{kg}/m}^{3}$. From Equation (24), $t_{s}=17.5209\;s$. The temperature period jump of 17.5209 s is taken for TCUs to vary temperature jump interval 0.1 °C, which ensures that the heat transfers from TCU to the center area uniformly and stably. To simplify the test steps, set $t_{P}=20\;s$. L3GD20 is chosen randomly and tested five times, and temperature is monitored using precise temperature measurement system with the accuracy of ±0.03 °C as well as the frequency of 10 Hz [18]. Based on that, a temperature experiment was designed whose flow chart is shown in Figure 8 and its steps are described as follows:

1 MEMS-gyro was closely attached to a metal shell as a module with thermal silicone grease and installed on the precise rate table. The temperature sensor of precise temperature measurement system was attached closely to the metal shell and measures the temperature of MEMS-gyro $T_{t}^{1}$. Get PC ready to record the data from MEMS-gyro in real time;
2 Start the rate table at the target $\omega_{s}$, while get PC ready to record the data from MEMS-gyro $D_{t}^{1}$;
3 Ambient temperature in thermal chamber goes down to −40 °C. After the data from MEMS-gyro and precise temperature measurement system are stable, start recording $T_{t}^{1}$ and $D_{t}^{1}$;
4 Ambient temperature in the thermal chamber goes up to 85 °C at a heating rate of 18 °C/h and stop the experiment when the data from MEMS-gyro and precise temperature measurement system keep stable for 1 h. Meanwhile, all the data during this period are recorded;
5 Repeat step (2) to step (4) 5 times and randomly select one group as the test data.

### 3.2. Parameter Identification for Modified TDE Compensation Model

**Figure 9:**
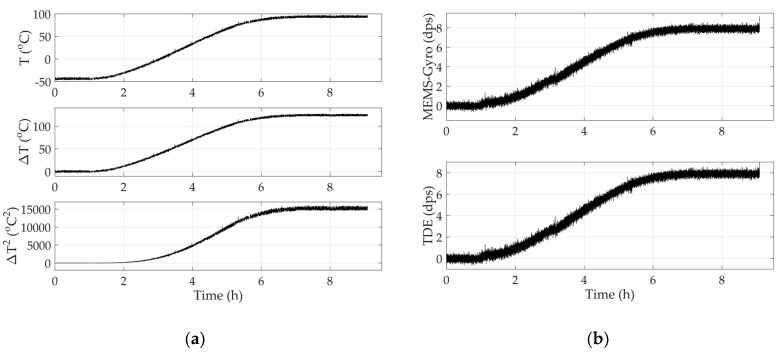
Experimental results of L3GD20. (**a**) ambient temperature and temperature variation as well as its square; (**b**) outputs of L3GD20 and its temperature drift error (TDE).

Under the premise of establishing modified TDE compensation model and testing TDE and TCQ, the accuracy of compensating TDE depends on parameter identification based on TDE and TCQ. To test MEMS-gyros conveniently, set $\omega_{s}=0$ and the output reference was 0°/s. Figure 9 shows one group of the experimental data of L3GD20 and its ambient temperature. Ambient temperature goes up from −40 °C to 85 °C, and its initial value keeps stable for a while which was set as the reference temperature. Temperature variation and its square as well as its TDE are also shown in Figure 9.

**Figure 10:**
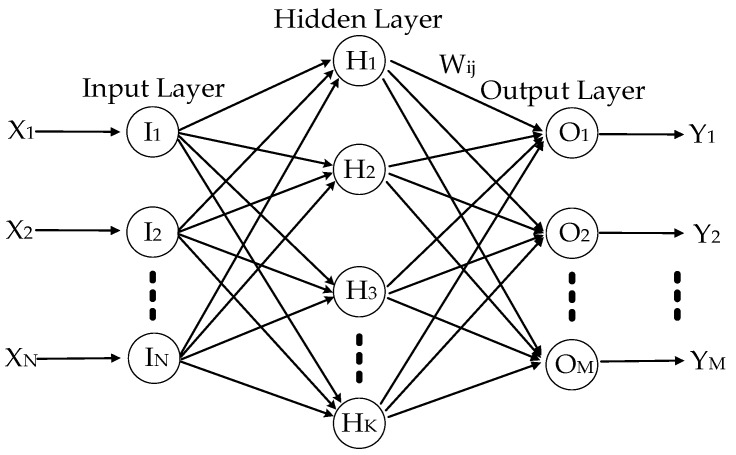
Structure of a radical basis function artificial neural network (RBF ANN).

As shown in Figure 9, when ambient temperature T varies, ambient temperature variation ΔT and its square $\Delta T^{2}$ vary in similar trend. With the help of ΔT and $\Delta T^{2}$, TDE have approximate trend. Based on that, it concludes that there is a complex nonlinearity among ΔT, $\Delta T^{2}$ and TDE. In addition, on the basis of Equation (16), it is necessary and essential to apply a nonlinear model with multiple inputs and multiple outputs which has high accuracy and remarkable real time to fit the complex nonlinearity. RBF ANN uses neurons as the basic computing units and neural layers as the basic computing framework. Neurons are distributed in different neural layers, including an input layer, a hidden layer and an output layer. The inputs are calculated and transmitted by neurons in three layers and the kernel functions in hidden layers. Finally, the outputs approximately approach to the targets. Figure 10 shows the structure of RBF ANN.

Where $X_{i}\left(i=1\cdots N\right)$ is the ith input of RBF ANN, and $Y_{i}\left(i=1\cdots M\right)$ is the ith output of RBF ANN, and $I_{i}\left(i=1\cdots N\right)$ is the ith neuron in input layer, and $H_{i}\left(i=1\cdots K\right)$ is the ith neuron in hidden layer, and $O_{i}\left(i=1\cdots M\right)$ is the ith neuron in output layer. Usually, Gaussian function is chosen as kernel function and the inputs are divided into several groups by kernel function, which is described by:

(26) $$\phi_{j}(x)=e^{-{\left\|x-c_{j}\right\|}^{2}/2\sigma_{j}^{2}}\;\;\;\;\;\;\;\;\;\;\;\;j=(1,2,\cdots ,K)$$

The most key point for RBF ANN is fixing the center $c_{j}$ and the width $\sigma_{j}$ of kernel function. As shown in Figure 10, input layer has N neurons and hidden layer has K neurons, as well output layer has M neurons. From Equation (26), $\phi_{j}\left(x\right)$ is the output of the jth neuron in the hidden layer, $c_{j}$ is the center vector of kernel function of the jth neuron in the hidden layer, x is a N-dimensional input vector, $\sigma_{j}$ is the width of Gauss function of the jth neuron in the hidden layer, $\left\|x-c_{j}\right\|$ is the distance between input vector and the center vector of Gauss function. Hence, the output of RBF ANN is shown:

(27) $$y_{j}=\sum_{j=1}^{K}W_{ij}\phi_{j}(x)\;\;\;\;\;\;\;\;\;\;\;\;j=(1,2,\cdots ,M)$$

where $y_{j}$ is the output of the jth neuron in the output layer, $W_{ij}$ is the weight between the jth neuron in the output layer and the jth neuron in the hidden layer. The sample set is divided to several groups by RBF ANN with Equation (26), and the outputs of neurons in the hidden layer is obtained. Using Equation (27), RBF ANN calculates the actual output with the weights and the outputs of neurons in the hidden layer. Comparing the actual output with the targets, the difference decides if $W_{ij}$, $c_{j}$ and $\sigma_{j}$ will be adjusted, and their adjusted magnitudes are shown as $\Delta W_{ij}$, $\Delta c_{j}$ and $\Delta \sigma_{j}$ respectively:

(28) $$\begin{array}{cc} \Delta W_{ij} & =\eta_{w}(d_{i}-y_{i})\phi_{j}(x)\\ \Delta c_{j} & =\eta_{c}\sum_{i=1}^{M}\left[(d_{i}-y_{i})W_{ij}\right]\frac{\left(x-c_{j}\right)}{\sigma_{j}^{2}}\phi_{j}(x)\\ \Delta \sigma_{j} & =\eta_{\sigma }\sum_{i=1}^{M}\left[(d_{i}-y_{i})W_{ij}\right]\frac{{\left\|x-c_{j}\right\|}^{2}}{\sigma_{j}^{3}}\phi_{j}(x) \end{array}$$

When RBF ANN is being trained, $W_{ij}$, $c_{j}$ and $\sigma_{j}$ will be adjusted separately with Equation (28). After being adjusted for several times, the actual outputs of RBF ANN meet the design requirement [18]. In addition, there are two advantages about RBF ANN as follows:

- RBF ANN can avoid local minimums. Owing that RBF ANN works on the basis of Gaussian functions, the current results are optimal in global scope, even in complex conditions like some flat areas where error gradient approximate to zero.
- According to Kolmogorov theorem, a three-layer forward network can approach any continuous function with any desired accuracy [18]. RBF ANN has the typical structure of input layer, hidden layer and output layer and is able to realize the nonlinearity in any accuracy. In addition—considering real time and the universality—the structure of the modified TDE compensation models should be as simple as possible. Hence, RBF ANN is good at improving the real time and the universality of TDE compensation models for MEMS-gyros.

Therefore, RBF ANN is the best choice to describe the nonlinearity among ΔT, $\Delta T^{2}$ and TDE accurately. Hence, Equation (16) can also be deduced as follows:

(29) $$\Delta E_{MEMS}=ANN_{RBF}(\Delta T,\Delta T^{2})$$

The parameter of the modified TDE compensation model should be identified as follows:

1 Two temperature experiments are carried out. The experimental data from one temperature experiments group are in training sample set and the other data are in verification sample set.
2 The sample set of TDE is obtained by subtracting the reference outputs of MEMS-gyros from their actual outputs in the training sample set. The sample set of ΔT is obtained by subtracting the reference temperature of MEMS-gyros from their temperature in the training sample set, and $\Delta T^{2}$ is obtained by multiplying itself.
3 RBF ANN is trained with ΔT and $\Delta T^{2}$ as the inputs and TDE as the output. Training will not stop until the differences between the outputs of RBF ANN and the corresponding TDE meet the design requirements.
4 The compensated results are obtained from subtraction between the outputs of RBF ANN and the corresponding outputs of MEMS-gyros.

**Figure 11:**
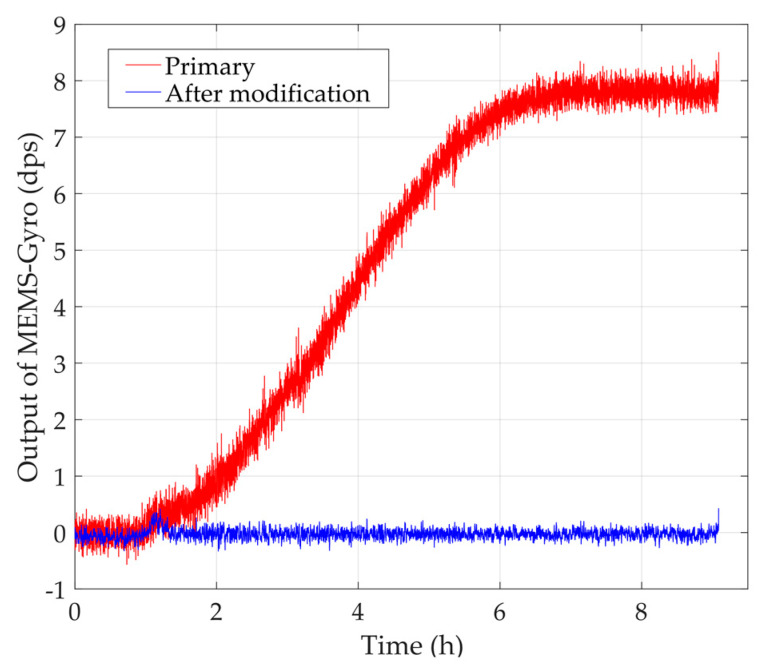
Primary outputs of MEMS-gyros and its compensated outputs.

Based on all the steps above, Equation (29) will be trained with the experimental data shown in Figure 9, and the parameters will be identified accurately. Then, the modified TDE compensation model will be checked again with verification sample set. Figure 11 shows the primary outputs of MEMS-gyros shown in Figure 9 and its compensated outputs.

According to Figure 11, the modified model can estimate TDE accurately and MEMS-gyros run stably while ambient temperature varies from −40 °C to 85 °C. The experimental results means ambient temperature almost has no impact on MEMS-gyros. Usually, MEMS-gyros’ performance is evaluated with the indexes, bias stability, angle random walk, angle rate random walk, quantization noise and rate ramp. Bias stability not only shows the dispersion degree between MEMS-gyros’ output and its reference, but also can illustrate the dispersion degree between their outputs before and after TDE compensation. Hence, bias stability is applied to evaluate the accuracy of the modified TDE compensation model and the stability of MEMS-gyro after compensation, which is shown as follows:

(30) $$BS=MSE(x-x^{\prime })$$

where x is the evaluated sample, $x^{\prime }$ is the reference of the evaluated sample, MSE is mean square error algorithm, and BS is bias stability between x and $x^{\prime }$. Bias stability is a remarkably intuitive index indicating the dispersion degree between the evaluated sample and its reference, which also shows the fluctuation of MEMS-gyro output after compensation. The smaller the bias stability, the smaller the dispersion degree between the evaluated sample and its reference is and the more accurately the modified model can estimate TDE. To illustrate the credibility and repetitiveness of the modified model, bias stabilities in five experiments before and after compensation are shown in Table 2.

From Table 2, the modified TDE compensation model effectively improve their stabilities and bias stabilities after compensation is about 4 orders of magnitude higher than before compensation. Hence, MEMS-gyro is unaffected by ambient temperature and the modified compensation model is able to decouple temperature dependence of Si-based materials remarkably.

## 4. Comparison of Test Results Before and After Modification

**Figure 12:**
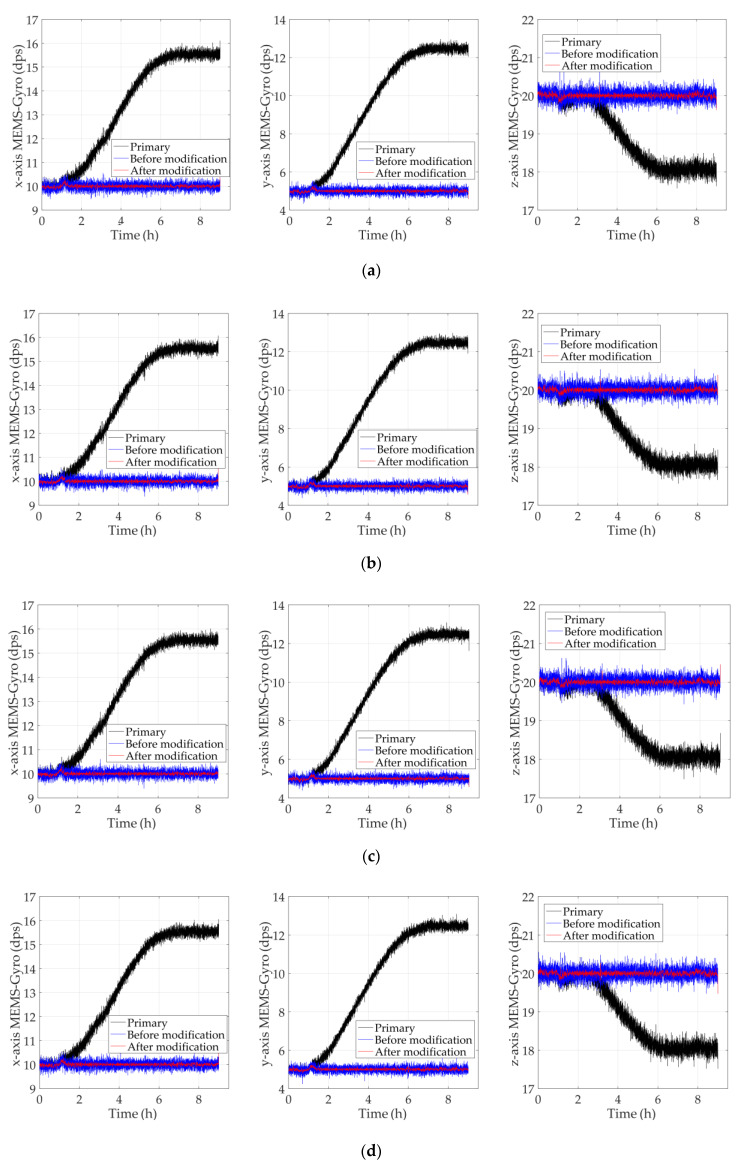

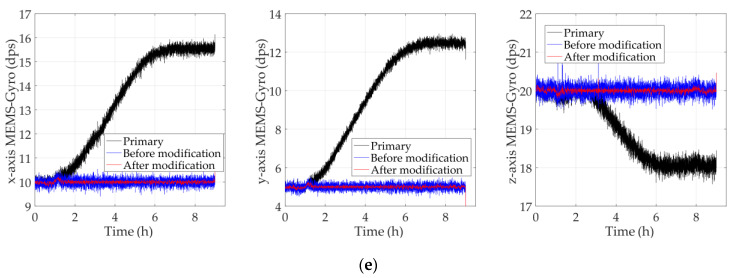
Comparison of the test results in five experiments. (**a**) 1st experiment; (**b**) 2nd experiment; (**c**) 3rd experiment; (**d**) 4th experiment; (**e**) 5th experiment.

To verify the performance and universality of the modified TDE compensation model further, MEMS-gyro I3G4250D manufactured by ST company is selected instead as the test object. Based on temperature experiment and parameter identification, the conventional and the modified models of MEMS-gyros in *x*-axis, *y*-axis and *z*-axis were established, and their compensation performances were verified and compared. In order to guarantee the universality of test results, the referenced angular velocity in *x*-axis, *y*-axis, *z*-axis were randomly set as $\omega_{ref}^{x}=10\;\mathrm{dps}$, $\omega_{ref}^{y}=5\;\mathrm{dps}$, $\omega_{ref}^{z}=20\;\mathrm{dps}$. Figure 12 shows the comparison of test results.

According to Equation (30), the evaluated formulas for the primary data, the data compensated by the conventional model and the data compensated by the modified model are shown as follows:

(31) $$\begin{array}{l} BS_{1}=MSE(k-k_{{}_{ref}}^{i})\\ BS_{2}=MSE\left[k\left(\Delta T\right)-k_{{}_{ref}}^{i}\right]\\ BS_{3}=MSE\left[k\left(\Delta T,\Delta T^{2}\right)-k_{{}_{ref}}^{i}\right] \end{array}$$

where k is the primary results of MEMS-gyros in *x*-axis, *y*-axis and *z*-axis; k(ΔT) is the test results compensated by the conventional model; $k\left(\Delta T,\Delta T^{2}\right)$ is the test results compensated by the modified model of MEMS-gyros; $k_{ref}^{i}\left(i=x,y,z\right)$ is the referenced angular velocity in *x*-axis, *y*-axis and *z*-axis. Bias stabilities in five experiments are shown as follows. To demonstrate the improvement between the primary data and the compensated results, performance improvement index is shown as below:

(32) $$P_{i}=\frac{BS_{i+1}}{BS_{i}}\;\;\;\;\left(i=1,2\right)$$

According to Figure 12, the modified models estimate and compensate TDE more accurately, which keeps MEMS-gyros running stably for a long time while ambient temperature varies. From Table 3, Table 4, Table 5, Table 6 and Table 7, bias stabilities of the modified models were significantly smaller than the conventional model, which is increased to about 10% of bias stability of the conventional model. Hence, the modified TDE compensation model decouple temperature dependence of Si-based materials more remarkably, which achieves the purpose of improving the environmental adaptability.

## 5. Conclusions

In this study, a modification of an MTEA-based temperature drift error compensation model of MEMS-gyros was presented. Using microstructure thermal effect analysis, the novel TCQ (temperature variation and its square) were extracted. Then, two key parameters for TDE precise test method, temperature jump interval and its period, were deduced with heat conduction analysis. The modified TDE compensation models were built based on input-modified RBF ANN and their performances were verified and compared. The experimental results show MEMS-gyros run stably and accurately while ambient temperature varies and bias stability was increased by more than one order of magnitude. Temperature dependence of Si-based materials was decoupled completely and the environmental adaptability of MEMS-gyros was improved even in diverse complicated conditions.

## Figures and Tables

**Table 1:** Comparison between resonator gyros.

.	Principle	Merits	Demerits
Tuning fork gyro	Coriolis effect and momentum conservation	Low cost Simple implementation	Low precision
Piezoelectric gyro	Coriolis effect and Piezoelectric effect	Long service life High reliability	Worse environmental adaptability
MEMS-gyro	Coriolis effect and capacitor	Low cost and small size Higher precision	Bad environmental adaptability
HR-gyro	Coriolis effect and precession effect of standing wave in radial vibration	Highest precision High bandwidth Strong overload	High cost Complex implementation

**Table 2:** Bias stabilities before and after compensation.

	BS_1_ (The Primary Data)	BS_2_ (After Compensation)	Improvement (BS_2_/BS_1_)
1st experiment	30.0402	7.882 × 10^−3^	2.6239 × 10^−4^
2nd experiment	29.3837	5.641 × 10^−3^	1.9199 × 10^−4^
3rd experiment	28.3816	5.615 × 10^−3^	1.9782 × 10^−4^
4th experiment	29.2548	5.630 × 10^−3^	1.9244 × 10^−4^
5th experiment	29.4201	5.758 × 10^−3^	1.9570 × 10^−4^

**Table 3:** Bias stabilities of the experimental data in 1st experiment.

	BS_1_	BS_2_	BS_3_	P_1_	P_2_	P_3_ = P_2_/P_1_
*x*-axis	15.3711	0.0055	3.885 × 10^−^^4^	3.594 × 10^−^^4^	2.528 × 10^−^^5^	7.03%
*y*-axis	29.2621	0.0077	6.201 × 10^−^^4^	2.617 × 10^−4^	2.119 × 10^−5^	8.09%
*z*-axis	1.5336	0.0075	4.571 × 10^−^^4^	4.889 × 10^−^^3^	2.980 × 10^−^^4^	6.09%

**Table 4:** Bias stabilities of the experimental data in 2nd experiment.

	BS_1_	BS_2_	BS_3_	P_1_	P_2_	P_3_ = P_2_/P_1_
*x*-axis	15.0619	0.0167	1.716 × 10^−^^3^	1.111 × 10^−^^3^	1.139 × 10^−^^4^	10.26%
*y*-axis	28.6982	0.0188	1.879 × 10^−^^3^	6.558 × 10^−4^	6.549 × 10^−5^	9.99%
*z*-axis	1.5975	0.0177	1.807 × 10^−^^3^	1.109 × 10^−^^2^	1.131 × 10^−^^3^	10.19%

**Table 5:** Bias stabilities of the experimental data in 3rd experiment.

	BS_1_	BS_2_	BS_3_	P_1_	P_2_	P_3_ = P_2_/P_1_
*x*-axis	15.7015	0.0174	1.654 × 10^−^^3^	1.109 × 10^−^^3^	1.054 × 10^−^^4^	9.49%
*y*-axis	29.1082	0.0180	1.737 × 10^−^^3^	6.183 × 10^−4^	5.969 × 10^−5^	9.65%
*z*-axis	1.5472	0.0184	1.904 × 10^−^^3^	1.190 × 10^−^^2^	1.231 × 10^−^^3^	10.34%

**Table 6:** Bias stabilities of the experimental data in 4th experiment.

	BS_1_	BS_2_	BS_3_	P_1_	P_2_	P_3_ = P_2_/P_1_
*x*-axis	15.6523	0.0179	1.870 × 10^−^^3^	1.146 × 10^−^^3^	1.195 × 10^−^^4^	10.42%
*y*-axis	29.0114	0.0187	1.813 × 10^−^^3^	6.449 × 10^−4^	6.249 × 10^−5^	9.69%
*z*-axis	1.4089	0.0180	1.740 × 10^−^^3^	1.275 × 10^−^^2^	1.235 × 10^−^^3^	9.69%

**Table 7:** Bias stabilities of the experimental data in 5th experiment.

	BS_1_	BS_2_	BS_3_	P_1_	P_2_	P_3_ = P_2_/P_1_
*x*-axis	15.3746	0.0172	1.749 × 10^−^^3^	1.117 × 10^−^^3^	1.138 × 10^−^^4^	10.18%
*y*-axis	29.4375	0.0186	1.757 × 10^−^^3^	6.319 × 10^−4^	5.970 × 10^−5^	9.45%
*z*-axis	1.5811	0.0185	2.038 × 10^−^^3^	1.171 × 10^−^^2^	1.289 × 10^−^^3^	11.01%
